# Inhibition of the NLRP3-inflammasome prevents cognitive deficits in experimental autoimmune encephalomyelitis mice via the alteration of astrocyte phenotype

**DOI:** 10.1038/s41419-020-2565-2

**Published:** 2020-05-15

**Authors:** Baohua Hou, Yahui Zhang, Peiyu Liang, Yuan He, Biwen Peng, Wanhong Liu, Song Han, Jun Yin, Xiaohua He

**Affiliations:** 1 Department of Pathophysiology, School of Basic Medical Sciences, Wuhan University, Wuhan, China; 2 Research Center for Medicine and Structural Biology, Wuhan University, Wuhan, China; 3 Hubei Provincial Key Laboratory of Developmentally Originated Disease, School of Basic Medical Sciences, Wuhan University, Wuhan, China; 4 Hubei Province Key Laboratory of Allergy and Immunology, School of Basic Medical Sciences, Wuhan University, Wuhan, China

**Keywords:** Multiple sclerosis, Neurodegenerative diseases, Multiple sclerosis, Neurodegenerative diseases, Inflammasome

## Abstract

Multiple sclerosis (MS) is a chronic disease that is characterized by demyelination and axonal damage in the central nervous system. Cognitive deficits are recognized as one of the features of MS, and these deficits affect the patients’ quality of life. Increasing evidence from experimental autoimmune encephalomyelitis (EAE), the animal model of MS, has suggested that EAE mice exhibit hippocampal impairment and cognitive deficits. However, the underlying mechanisms are still unclear. The NLRP3 inflammasome is a key contributor to neuroinflammation and is involved in the development of MS and EAE. Activation of the NLRP3 inflammasome in microglia is fundamental for subsequent inflammatory events. Activated microglia can convert astrocytes to the neurotoxic A1 phenotype in a variety of neurological diseases. However, it remains unknown whether the NLRP3 inflammasome contributes to cognitive deficits and astrocyte phenotype alteration in EAE. In this study, we demonstrated that severe memory deficits occurred in the late phase of EAE, and cognitive deficits were ameliorated by treatment with MCC950, an inhibitor of the NLRP3 inflammasome. In addition, MCC950 alleviated hippocampal pathology and synapse loss. Astrocytes from EAE mice were converted to the neurotoxic A1 phenotype, and this conversion was prevented by MCC950 treatment. IL-18, which is the downstream of NLRP3 inflammasome, was sufficient to induce the conversion of astrocytes to the A1 phenotype through the NF-κB pathway. IL-18 induced A1 type reactive astrocytes impaired hippocampal neurons through the release of complement component 3 (C3). Altogether, our present data suggest that the NLRP3 inflammasome plays an important role in cognitive deficits in EAE, possibly via the alteration of astrocyte phenotypes. Our study provides a novel therapeutic strategy for hippocampal impairment in EAE and MS.

## Introduction

Multiple sclerosis (MS) is a chronic disease that is characterized by demyelination and axonal damage in the central nervous system (CNS). Although motor impairment is the main disease process in MS, increasing studies have indicated that cognitive deficit is a common concomitant symptom of MS in both early and late stages^1–3^. MS-related cognitive deficits affect many aspects of daily life, including the participation in social activities, driving ability, and employment status^4^. Experimental autoimmune encephalomyelitis (EAE) mice are used to model the disease progression of MS and mirror MS-like pathology. EAE mice also exhibit cognitive deficits and significant disruption in the structural integrity of synapses^5–7^. Moreover, glial cells are necessary for hippocampal synaptic alterations and contextual learning-memory impairment in EAE^6,8^. However, the underlying mechanism remains largely unknown.

It has been reported that the immune system and the CNS dynamically interact under pathological conditions and neuroinflammation has potential to influence long-term synaptic plasticity, the basis of memory^9^. The NLRP3 inflammasome is comprised of the NLR family, pyrin domain containing 3 (NLRP3), apoptosis-associated speck-like protein containing a carboxyterminal CARD (ASC), and pro-caspase-1. The NLRP3 inflammasome is considered as the key contributor of neuroinflammation, and the activated NLRP3 inflammasome processes pro-IL-1β and pro-IL-18 to produce mature IL-1β and IL-18, respectively^10^. In the CNS, the NLRP3 inflammasome, IL-1β and IL-18 are found in microglia^11–13^ and take part in many diseases of the nervous system^14^. In MS lesions, the expression of caspase-1, IL-1β, and IL-18 is elevated^15,16^, which suggests the involvement of the NLRP3 inflammasome in MS pathogenesis. Moreover, Nlrp3^−/−^ mice are resistant to EAE and exhibit less immune cell infiltration^17^.

Astrocytes are the most abundant glial cells and are vital for neuronal network regulation. Reactive astrocytes exhibit A1 and A2 phenotypes. A1 astrocytes highly express many neurotoxic genes, such as *H2, T23*, *ligp1*, and *Fkbp5*, while A2 astrocytes highly express many neuroprotective factors. A1 astrocytes are presented in neuroinflammatory and neurodegenerative diseases, such as Alzheimer’s disease (AD), Huntington’s disease (HD), Parkinson’s disease (PD), amyotrophic lateral sclerosis (ALS), and MS^18^. In MS lesions, A1 astrocytes are typically closely associated with activated microglia, which suggests that A1 astrocytes might be induced by activated microglia^18^. However, it is unclear whether the NLRP3 inflammasome in microglia is involved in astrocyte phenotype alteration.

Our present study was designed to investigate the cognitive deficits in EAE mice and the effect of the NLRP3 inflammasome on astrocyte phenotype alteration.

## Materials and methods

### Animals

Six-week-old female C57BL/6 mice were obtained from the Wuhan University Center for Animal Experiment/ABSL-3 Laboratory. All experimental procedures complied with the Committee on the Ethics of Animal Experiments of Wuhan University (China) (permit number: 2017083). The mice were grouped and kept under standard laboratory conditions (a 12-h light/dark cycle with an average room temperature of 25 °C and a relative humidity of 55–60%). The mice were provided food and water available ad libitum during the study.

### EAE induction and treatment

The EAE model was induced as previously described^19^. Mice were anaesthetized and subcutaneously immunized with 200 μl of 100 μg myelin oligodendrocyte glycoprotein (MOG_35–__55_, Wuhan Haode Peptide, China) in incomplete Freund’s adjuvant (Sigma Aldrich, USA) containing 200 μg mycobacterium tuberculosis (strain H37Ra; Difco, USA) on day 0. Pertussis toxin (PTX; List Biologicals, USA) (200 ng) was administered intraperitoneally (i.p.) on day 0 and 2. EAE scores were assessed daily for clinical signs of EAE mice in a blinded fashion as previously described^20^. EAE scores were evaluated as followed: 0.5, partial tail limpness; 1, tail limpness; 1.5, reversible impaired righting reflex; 2, impaired righting reflex; 2.5, paralysis of one hindlimb; 3, paralysis of both hindlimbs; 3.5, paralysis of both hindlimbs and one forelimb; 4, hindlimb and forelimb paralysis; 5, death. Animals were scored daily by two independent investigators in a blind fashion.

For the behavioral experiments, the mice were randomly divided into three groups (*n* = 16 in each group): (1) the control group (Ctrl), mice received saline; (2) the EAE group (EAE), mice were received immunization; (3) the EAE + MCC950 group (EAE + MCC950), EAE mice were received MCC950 (a specific NLRP3 inhibitor; Medchem Express, China). Recent studies have shown that MCC950 can cross the blood brain barrier and act on the central area directly^21–23^. According to the previous research^24^, MCC950 was administered intraperitoneally injection to mice (10 mg/kg) at induction of the disease, day 0, 1, and 2 and every 2 days thereafter.

### Cell culture and treatment

Primary astrocytes were prepared as previously reported^25^. Cerebral cortices were dissected from newborn mice and digested in 0.25% trypsin for 8 min at 37 °C. After enzyme treatment, the cells were dispersed and collected by trituration through a pipette, then the cells were cultured in DMEM F12 supplemented with 10% FBS, 50 U/ml penicillin, and 50 μg/ml streptomycin for 12 days. To purify the astrocytes, flasks were continuously shaken overnight to remove microglia and oligodendrocytes. Under this condition, over 99% cells were GFAP-positive confirmed by immunocytochemistry.

As serum is known to induce astrocyte activation^26,27^, astrocytes were washed once with HBSS and subsequently cultured in serum-free medium (Sato’s serum-free medium, 10 μg/ml insulin, 100 μg/ml transferrin, 300 μg/ml bovine serum albumin, 16 μg/ml putrescine, 400 ng/ml thyroxine, 300 ng/ml tri-iodo-thyronine, 60 ng/ml progesterone, 40 ng/ml sodium selenite, and 1 mM Glutamax in low glucose DMEM with pyruvate, Life Technologies, USA) for 5 days before treatment. Then, primary astrocytes were treated with PBS, recombinant mouse IL-1β (100 ng/ml and 500 ng/ml, R&D Systems, USA) or recombinant mouse IL-18 (100 ng/ml and 500 ng/ml, R&D Systems, USA) for 24 h. To study the effect of astrocyte-derived factors on neuron, conditioned media from astrocytes were used as control ACM and IL-18 induced ACM, respectively. Additionally, control ACM and IL-18 induced ACM were collected with a protease inhibitor (Sigma Aldrich) and concentrated with a TCA Protein Precipitation Kit (Sangon Biotech, China). Total protein was collected for western blotting.

Primary hippocampal neurons were isolated from C57BL/6 mice as previously described with slight modifications^28^. Briefly, primary hippocampal neurons were prepared from postnatal day 0 pups and cultured in neurobasal medium (Thermo Scientific, USA) supplemented with 2 mM l-glutamine (Thermo Scientific), 2% B27 (Thermo Scientific), 50 U/ml penicillin (Thermo Scientific, USA), and 50 μg/ml streptomycin (Thermo Scientific) on poly-l-lysine-coated circular glass coverslips (12-mm diameter). The culture medium was changed twice per week, and immunofluorescence staining for microtubule-associated protein 2 (MAP2, 1:500, GeneTex, USA, GTX634473) was performed to verify the purity of the hippocampal neuronal population. After the hippocampal neurons were cultured in neurobasal medium for 10 days, all the culture medium was replaced with different ACMs and maintained for 3–5 days. For western blot and immunostaining, the samples were collected at the 4th day. For the electrophysiological experiments, the recording started at the 3rd day, and the whole electrophysiological experiments continued from the 3rd day to the 5th day. Hippocampal neurons were divided into three groups (*n* = 6 for each group): (1) the control ACM group, neurons cultured in control ACM; (2) the IL-18 induced ACM group, neurons cultured in IL-18 induced ACM; and (3) the IL-18 induced ACM + SB209157 (SB) group, neurons incubated with the C3a receptor antagonist SB290157 (10 μM; MedChem Express) 60 min before IL-18 induced ACM stimulation.

In order to investigate the effect of residual recombinant IL-18 in ACM on the neurons. Primary hippocampal neurons were directly treated with PBS or recombinant mouse IL-18 (100 ng/ml and 500 ng/ml, R&D Systems) for 24 h. Total protein was collected for western blotting.

### Behavioral experiments

The Morris water maze test was performed as described previously^29^. Spatial memory testing was conducted in a pool consisting of a circular tank (Ø1 m) filled with opaque water maintained at 23 ± 1 °C. The water basin was dimly lit and was surrounded by a white curtain. The maze was virtually divided into four quadrants, with one quadrant containing a hidden platform (15 × 15 cm) hidden 1.5 cm below the water surface. For each trial, the mice started from one of the four quadrants facing the wall of the pool and were trained to find the platform for 60 s. If a mouse did not reach the platform in the allotted time, it was placed onto the platform manually for 15 s. The mice were trained for four trials per day for 6 consecutive days. For the spatial probe trials, which were conducted 24 h after the last training session, the platform was removed and the mice were allowed to swim for 60 s. All movements were recorded by a computerized tracking system that calculated the distance traveled and latency required to reach the platform (SMART, Panlab Harvard Apparatus, USA). All data were recorded and analyzed in a blind fashion.

The fear conditioning test was performed as described previously^8^. The mice were placed in an arena (context) with a grid floor conducted to a Multi Conditioning System (Startle and Fear Combined System, DL Naturegene Life Sciences, China). The first activity test was performed three times with light and noise conditions (29 s) and was followed by inescapable electroshocks (0.6 mA, 1 s) delivered at intervals of 1 min. On the following day (for the 24-h test), the mice were placed in the same arena with the absence of electroshocks for the mnemonic fear expression test, which comprised four tests divided into 1 min intervals. The main measure in each test was the percentage of time spent freezing per interval, with freezing defined as an episode during which no movement was detected for at least 2 s. For the conditioning test, the mean percentage of time freezing was calculated for each pair of consecutive intervals between electroshocks (0.5–2.0, 2.5–4.0, and 4.5–6.0 min); for the expression test, the mean percentage of time spent freezing was calculated for each trio of consecutive intervals (0.5–2.0, 2.5–4.0, 4.5–6.0, and 6.5–8.0 min). All data were recorded and analyzed in a blind fashion.

### Tissue preparation

Fresh tissues were extracted from the hippocampus and frozen immediately for biochemical analysis at the peak phase (day 19) and the late phase (day 46) of EAE mice. For the pathological and immunohistochemistry test, mice were deeply anaesthetized and transcardially perfused with 50 ml of phosphate-buffered saline (PBS) followed by 50 ml of 4% paraformaldehyde (PFA). Perfusion-fixed brains and spinal cords were further fixed in PFA overnight at 4 °C.

### Hematoxylin and eosin staining

PFA-fixed brains were processed for embedding in paraffin and cut into 4 μm sections. After deparaffinization and hydration, the sections were stained with hematoxylin-eosin (H&E) and photographed with an Olympus AH-2 light microscope (×200; Olympus, Japan).

### Luxol fast blue staining

PFA-fixed spinal cords were processed for embedding in paraffin and cut into 4 μm sections. After deparaffinization and hydration, the sections were stained with Luxol fast blue (LFB) and photographed with an Olympus AH-2 light microscope (×200; Olympus). Demyelination score was evaluated using the following scale described by Wraith et al.^30^ (0 = no demyelination; 1 = a few, scattered naked axons; 2 = small groups of naked axons; 3 = large groups of naked axons; and 4 = confluent foci of demyelination).

### Nissl staining

To assess neuronal loss, we performed Nissl staining. Sections (4 μm) were deparaffinized, hydrated, and stained with toluidine blue solution (Boster Biotech, China). The number of surviving intact neurons was counted under high magnification using an Olympus AH-2 light microscope (×200; Olympus). Five sections from each group were analyzed.

### Golgi staining

Fixed brains were washed with PBS several times and incubated in Golgi-Cox solution (Servicebio, China) for 14 days. Then, the brains were incubated for 2 days in 30% fresh sucrose in PBS and were sliced at a thickness of 100 μm using a vibratome (VT1000S; Leica, Germany). The slices were collected for silver staining. The images were photographed with an Olympus AH-2 light microscope (Olympus). For Scholl analysis, at least 10 neurons from hippocampal DG region of each group were analyzed.

### Transmission electron microscopy

Lumbar spinal cord was fixed by immersion in 2.5% glutaraldehyde for 2 h, and post-fixed in 1% OsO_4_ to increase the membranes contrast. Subsequently, the samples were dehydrated in a graded series of ethanol and propyleneoxide, and embedded in Epon. Thin sections of the Epon-embedded blocks were stained with a solution of uranyl acetate and lead citrate. The stained sections were observed by Transmission Electron Microscope (TEM, Hitachi, Tokyo, Japan) at 80 kV. For analysis of myelin pathology, the myelin thickness was measured using ImageJ software (version 1.46r, USA).

### Immunohistochemistry

Fixed brains and spinal cords were paraffin-embedded and cut into 4 μm sections. After antigen retrieval, the sections were blocked with 10% normal goat serum in PBS and stained with anti-Iba1 (1:500, Proteintech, China, 10904-1-AP) antibody, CD45 (1:200, GeneTex, GTX65913), MBP (1:200, Abcam, ab40390), PSD95 (1:500, Cell Signaling Technology, D74D3), or synapsin I (1:500, Cell Signaling Technology, D12G5). Then the sections were incubated with a horseradish peroxidase (HRP)-conjugated anti-rabbit antibody (1:500, Abbkine, China). The sections were developed using DAB Peroxidase Substrate (Beyotime Biotechnology, China). The sections were photographed with an Olympus AH-2 light microscope (×200; Olympus). The number of visualized cells was measured with ImageJ software.

Fixed brains were washed four times in PBS and incubated for 1.5 days in 30% fresh sucrose in PBS at 4 °C. Then, 20-μm-thick frozen brain slices were cut and used for immunofluorescence labeling. After being blocked with 5% bovine serum albumin (BSA) in PBS, the slices were incubated with mouse anti-ASC (1:50, sc-271054, Santa Cruz Biotechnology, USA), rabbit anti-Iba1 (1:200, Proteintech), rabbit anti-GFAP (1:200, 16825-1-AP, Proteintech, China), goat anti-C3d (1:200, AF2655, R&D System, USA), or mouse anti-MAP2 (1:500, GeneTex) antibodies overnight at 4 °C. Then the slices were incubated with DyLight 549-conjugated duck anti-mouse IgG (H + L), DyLight 488-conjugated duck anti-rabbit IgG (H + L) or DyLight 549-conjugated duck anti-goat IgG (H + L) (1:400, Abbkine) secondary antibodies for 1 h at 37 °C. The slices were washed in PBS and incubated with DAPI (300 nM, Sigma Aldrich) to label nuclei. Images were obtained using a confocal microscope (Leica-LCS-SP8-STED, Germany).

Fixed primary astrocytes or hippocampal neurons were washed, permeabilized, blocked, and incubated with goat anti-C3d (1:200, R&D System), p65 (1:1000, 10745-AP, Proteintech, China), mouse anti-MAP2 (1:500, GeneTex), rabbit anti-PSD95 (1:400, GTX133091, GeneTex, USA), or rabbit anti-synapsin I (1:200, Cell Signaling Technology) antibodies overnight at 4 °C. On the following day, the coverslips were incubated with DyLight 549-conjugated duck anti-goat IgG (H + L), DyLight 488-conjugated duck anti-goat IgG (H + L), DyLight 549-conjugated duck anti-mouse IgG (H + L), or DyLight 488-conjugated duck anti-rabbit IgG (H + L) (1:400, Abbkine) for 1 h at 37 °C. Next, coverslips were washed in PBS and incubated with DAPI (300 nM, Sigma Aldrich) to label the nuclei. Images were obtained using a confocal microscope (Leica-LCS-SP8-STED).

### Western blotting

Fresh samples were extracted from hippocampal tissues or cultured cells. They were homogenized on ice with radioimmunoprecipitation assay (RIPA; Biosharp, China) buffer containing phenylmethanesulfonyl fluoride (PMSF; Biosharp, China) and phosphatase inhibitors (Roche, Switzerland). The protein content was determined by a BCA Protein Quantitation Kit (Thermo Scientific). Protein samples were separated by 10–12% sodium dodecyl sulfate-polyacrylamide gel electrophoresis (SDS-PAGE). After electrophoresis, the proteins were transferred to a polyvinylidene fluoride membrane (PVDF; Millipore, UK). The membranes were blocked with 5% BSA for 2 h at room temperature. The membranes were then incubated overnight at 4 °C with the following primary antibodies: anti-PSD95 (1:1000, Cell Signaling Technology); anti-synapsin I (1:1000, Cell Signaling Technology); anti-C3d (1:1000, R&D System); anti-S100A10 (1:1000, AF2377, R&D System, USA); anti-ASC (1:1000, AL177, AdipoGen, Switzerland); anti-IL-1β (1:1000, ab9722, Abcam, USA; GTX74034, GeneTex; DF6251, Affinity Biosciences, USA); anti-IL-18 (1:1000, abs135772, Absin, China); anti-Caspase-1 p10 (1:1000, ab179515, Abcam); anti-CD68 (1:1000, ab53444, Abcam,); anti-NLRP3 (1:1000, MAB7578, R&D System); anti-GFAP (1:1000, Proteintech); anti-p65 (1:1000, Proteintech); anti-phospho-NF-κB p65 (Ser536) (1:1000, 93H1, Cell Signaling Technology, USA); and anti-Caspase-3 (1:1000, 19677-1-AP, Proteintech, China). The membranes were washed with Tris-buffered saline containing 0.2% Tween-20 (TBST). Then, the membranes were incubated with an HRP-conjugated secondary antibody for 2 h (1:5000, Proteintech) at room temperature. The membranes were washed again with TBST. Finally, the protein band signals were developed by enhanced chemiluminescence (ECL; Thermo Scientific). The band densities were measured by densitometry and quantified using ImageJ software. β-actin (1:10,000, Proteintech) was used as a control.

### Enzyme linked immunosorbent assay

IL-18 concentration of different ACMs was measured using Enzyme linked immunosorbent assay (ELISA; Elabscience, China) according to the manufacturer’s instructions.

### Quantitative real-time PCR

Total RNA was isolated and purified from cultured astrocytes using TRIzol reagent (Invitrogen, USA). RNA extracts (2 μg) were reverse transcribed into complementary DNA (cDNA) using a Revert Aid First Strand cDNA Synthesis Kit (Thermo Scientific). Quantitative real-time PCR (qRT-PCR) was performed with SYBR Green Real-Time PCR Master Mix (Toyobo, Japan) according to the manufacturer’s instructions. The primers were as follows: *Cd109*: F: CACAGTCGGGAGCCCTAAAG, R: GCAGCGATTTCGATGTCCAC; *Fkbp5*: F: TATGCTTATGGCTCGGCTGG, R: CAGCCTTCCAGGTGGACTTT; *H2-T23*: F: GGACCGCGAATGACATAGC, R: GCACCTCAGGGTGACTTCAT; *Iigp1*: F: GGGGCAATAGCTCATTGGTA, R: ACCTCGAAGACATCCCCTTT; *S100a10*: F: CCTCTGGCTGTGGACAAAAT, R: CTGCTCACAAGAAGCAGTGG; *Emp1*: F: GAGACACTGGCCAGAAAAGC, R: TAAAAGGCAAGGGAATGCAC; and *GAPDH*: F: TCGCTCCTGGAAGATGGTGAT, R: CAGTGGCAAAGTGGAGATTGTTG. Expression was analyzed and statistics were performed using the Bio-Rad CFX96 Real-Time PCR System (Bio-Rad Laboratories, USA). Relative expression was determined using the 2^-ΔΔCt^ method.

### In vitro electrophysiological experiments

Electrophysiological experiments were performed in the whole-cell model at room temperature. The standard external solution contained 150 mM NaCl, 5 mM KCl, 1 mM MgCl_2_, 2 mM CaCl_2_, 10 mM HEPES, and 10 mM glucose. The pH was adjusted to 7.40 with 1.0 mM NaOH. The osmolarity was adjusted to 300–310 mOsm. For the recording of evoked action potentials (APs) in hippocampal neurons, the patch pipette was filled with internal solutions containing 145 mM KCl, 5 mM NaCl, 10 mM HEPES, 5 mM EGTA, 4 mM MgATP, and 0.3 mM Na_2_GTP with an adjusted pH of 7.20 and an adjusted osmolarity of 270–290 mOsm. APs were evoked by injection with different currents in steps of 10 pA, and the frequency was calculated. To record spontaneous excitatory postsynaptic currents (sEPSCs) from hippocampal neurons, the patch pipette was filled with internal solutions containing 145 mM CsCl, 5 mM NaCl, 10 mM HEPES, 5 mM EGTA, 4 mM MgATP, and 0.3 mM Na_2_GTP with an adjusted pH of 7.20 and an adjusted osmolarity of 270–290 mOsm. Picrotoxin (PTX; 100 μM, Tocris, USA) was added to the extracellular solution to block action potentials and GABA_A_ receptor-mediated inhibitory currents. The cell membrane potential was held at −60 mV, and the series resistance was compensated by ≥70%. APs and sEPSC were recorded using an Axopatch 700B amplifier and a Digidata 1550 interface (Axon Instruments, USA) and analyzed with Clampfit software (Axon Instruments) and Minianalysis program (Synaptosoft, USA).

### Data analysis

All data are expressed as the mean ± standard error of the mean (SEM). Statistical analysis was performed using GraphPad Prism software (version 6, USA). Statistical differences among the three groups were determined using one-way ANOVA with the Newman–Keuls test. Statistical differences between two groups were analyzed using Student’s *t*-test. For all analyses, statistical significance is denoted as *P < 0.05, **P < 0.01, or ***P < 0.001.

## Results

### NLRP3 inflammasome formation in the hippocampus of EAE mice

**Fig. 1:**
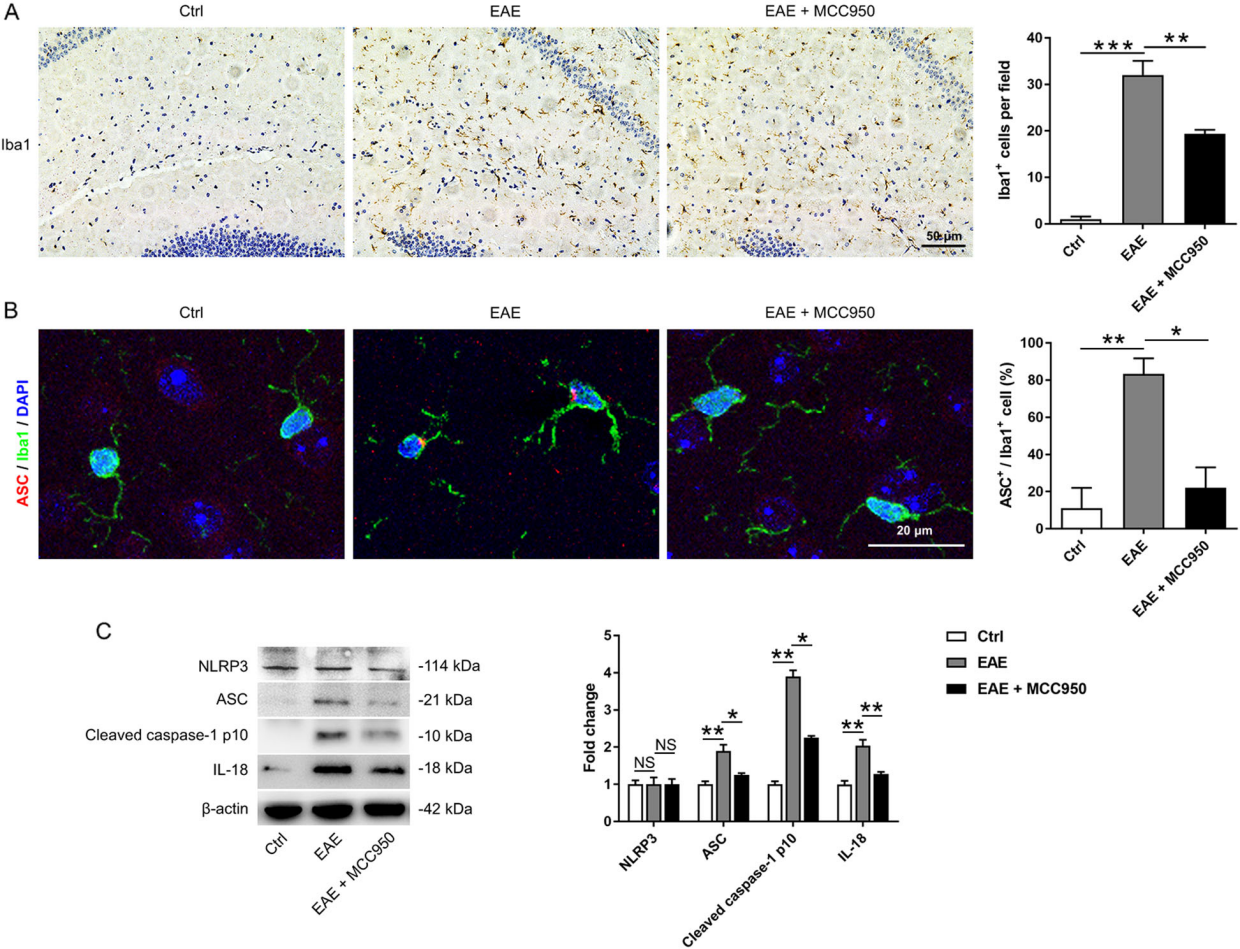
NLRP3 inflammasome formation in the hippocampus of EAE mice. **a** Immunohistochemistry for Iba1 in the hippocampus of each group. Field: ×200 200 μm. **b** Immunofluorescence for ASC (red), Iba1 (green), and DAPI (blue) in the hippocampus of each group. **c** Western blots and densitometric analysis for NLRP3, ASC, cleaved caspase-1 p10, and IL-18. β-actin was used as an internal control. The data are mean ± SEM, *n* = 6, *P < 0.05, **P < 0.01, ***P < 0.001.

It has been demonstrated that the hippocampal microglia in EAE mice are persistently activated^31^. However, few studies have investigated the NLRP3 inflammasome in the EAE brain. In our experiment, we used MCC950, a selective inhibitor of NLRP3, to investigate the activation of the NLRP3 inflammasome in the hippocampus of EAE mice at the peak phase (day 19). First, we examined the activation of microglia by immunohistochemistry and western blotting. We found that microglia were significantly activated in the EAE group, and MCC950 reduced the number of activated microglia (Figs. 1a and S1C). Because Iba-1 positive cells could also be macrophages, we performed immune-stained with CD45 (a specific macrophage marker). Macrophages are characterized by high CD45 expression, while microglia are characterized by low CD45 expression. No positive-signal of CD45 was found in the cortex or hippocampus of the EAE mice, which indicated that the Iba1-positive cells in the hippocampus of EAE mice might belong to resident microglia rather than macrophages infiltrating from peripheral (Fig. S2). Immunofluorescence with an antibody that recognizes the inflammasome component ASC was also performed. ASC speck formation was detected in the hippocampus of EAE mice, while pretreatment with MCC950 reduced ASC speck formation (Fig. S1A, B). In addition, we also quantified the co-staining of ASC and Iba1. We found that the number of ASC-positive microglia was increased in the EAE mice, and this increase was blocked by MCC950 (Fig. 1b). Key factors in the inflammasome pathway were also assessed. The levels of pro-IL-1β, IL-18, ASC, and cleaved caspase-1 p10 were increased, and pretreatment with MCC950 decreased the expression of these proteins (Figs. 1c and S1C). We also found that the levels of NLRP3 had not significant difference between the three groups (Fig. 1c). These results show that microglia and NLRP3 inflammasome formation are activated in the hippocampus of EAE mice.

### Inhibition of NLRP3 inflammasome improves cognitive deficits in EAE mice

**Fig. 2:**
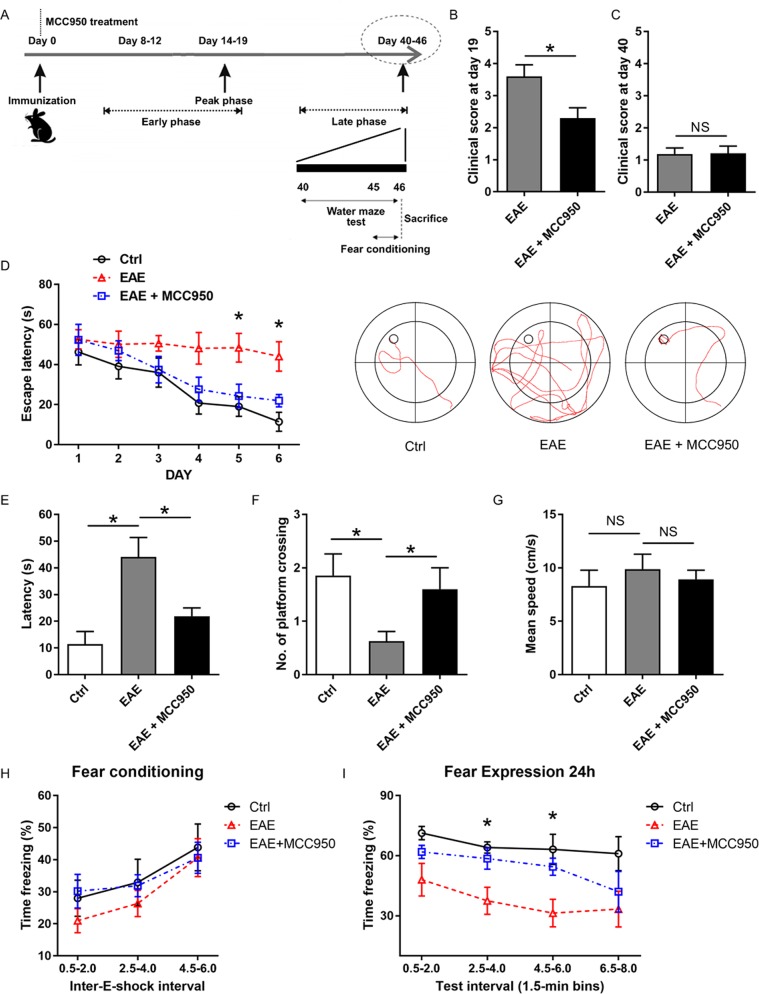
Effects of the NLRP3 inflammasome on behavioral performance in EAE mice. **a** A schematic illustration of EAE progression. **b** Clinical scores of C57BL/6 mice treated with PBS or MCC950 on day 19 after EAE induction. **c** Clinical scores of C57BL/6 mice treated with PBS or MCC950 on day 40 after EAE induction. **d** The training track of the mice in the Morris water maze test was recorded and analyzed. The escape latency to find the hidden platform during the 6 consecutive days of the training phase (left). Representative paths of the mice during their search for the hidden platform (right). **e** The time to reach the target during the probe trials. **f** The number of crossings over the target location during the probe trials. **g** The swimming speed of the three groups. The mice were first exposed to contextual fear conditioning test (**h**) and then to the fear expression test 24 h later (**i**). The data are the mean ± SEM; *n* = 15, *P < 0.05.

EAE mice exhibit motor disabilities, which are similar to the onset phase of MS patients^32^. In our experiment, EAE was characterized by an early phase and a late phase. Behavior performance was evaluated in the late phase (40 days after immunization) (Fig. 2a). First, we investigated the effects of MCC950 on EAE severity. As shown in Figs. 2b, S3A, and S4, treatment with MCC950 significantly reduced the severity of EAE during the peak phase (19 days after immunization). Twenty days after immunization, the motor ability of EAE mice became recovered, which showed the beginning of the late phase^32^. At the 40th day, no significant difference was found in clinical scores and the myelin thickness between the EAE group and the EAE + MCC950 group (Figs. 2c, S3A, and S5), and this time point was suitable to conduct behavioral experiments. Moreover, no significant difference was found in these three groups (Fig. S3B).

During the late phase, the Morris water maze test was performed to investigate the effect of MCC950 treatment on EAE-induced cognitive deficits. Compare with the control mice, EAE mice showed prolonged escape latency during the six consecutive training phases (Fig. 2d). In the probe trials, EAE mice showed extended latency to find the platform and a decreased number of platform crossings compared with the control group (Fig. 2e, f). However, MCC950-treated EAE mice were largely protected from spatial memory impairment. We also found that the swimming speed was not significantly different in these three groups (Fig. 2g).

The fear conditioning test was performed to further evaluate the cognition of EAE mice. On day 44, the fear conditioning test was conducted. All three groups learned proficiently and acquired conditioned fear during the sessions, while EAE mice showed a trend towards less freezing (Fig. 2h). After 24 h, long-term contextual memory was measured, and the EAE mice showed a trend toward decreased freezing compared to the control group. However, the EAE + MCC950 group showed similar fear expression as that shown in the control group (Fig. 2i). These data indicate that the inhibition of NLRP3 inflammasome ameliorates the EAE-induced cognitive deficits.

### NLRP3 inflammasome activation contributes to hippocampal pathology in EAE mice

**Fig. 3:**
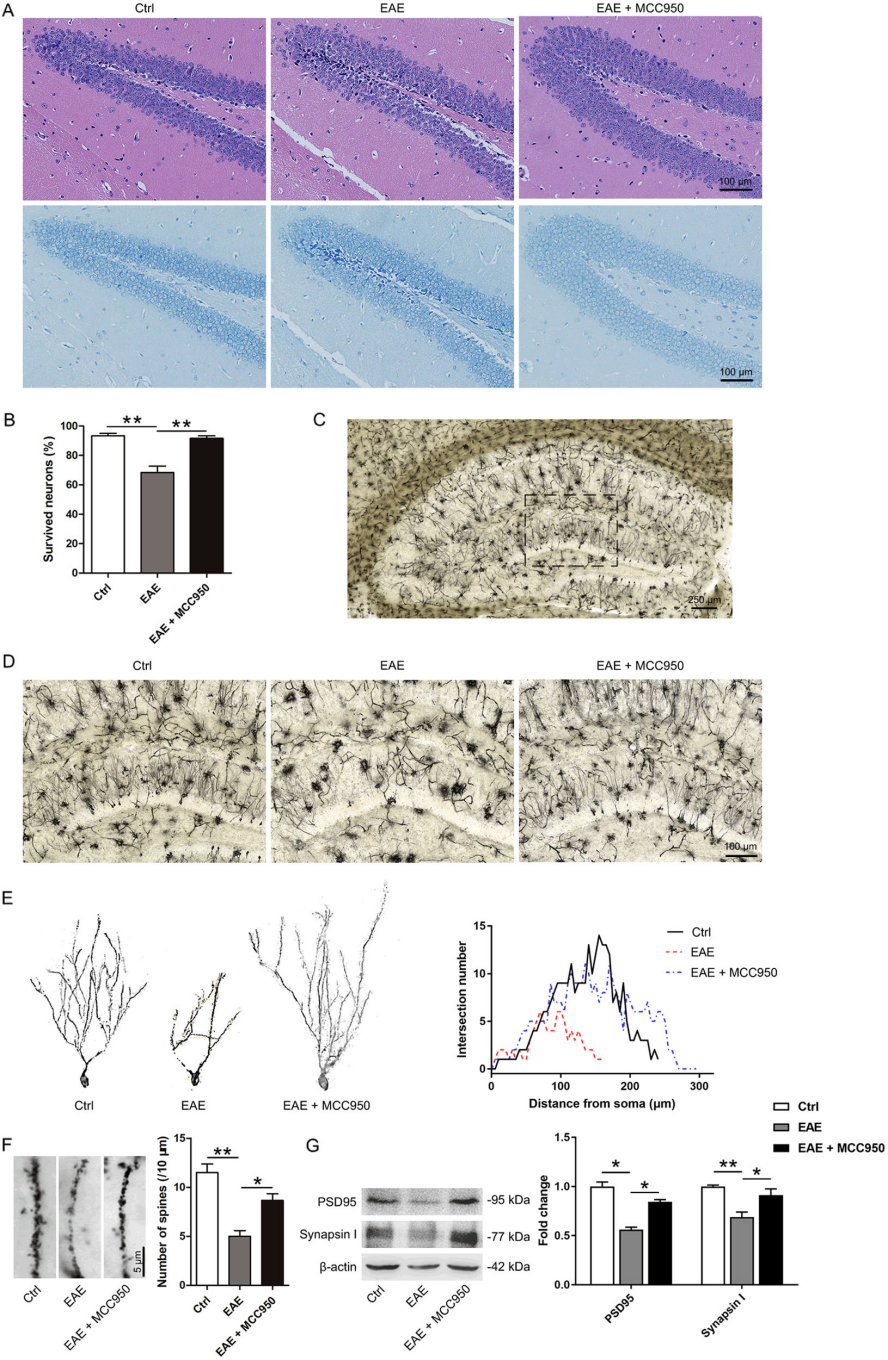
NLRP3 inflammasome activation contributes to hippocampal pathology in EAE mice. **a** Representative Nissl and H&E staining of the hippocampal DG region. **b** The quantitative analysis of intact hippocampal DG neurons. **c** Representative Golgi staining of the hippocampal DG region. **d** Representative magnified images of Golgi staining in the DG region of the three groups. **e** A graphical drawing showing neuron. At least 10 neurons from 6 mice per group were analyzed by Sholl analysis. **f** Representative images and the quantification of the spine number in the different groups. **g** Western blots and densitometric analysis for synapsin І and PSD95. β-actin was used as an internal control. The data are the mean ± SEM, *n* = 6 biologically independent animals, *P < 0.05, **P < 0.01.

The Morris water maze test and fear conditioning test are memory tasks involving the hippocampus. Therefore, we performed H&E and Nissl staining to assess the effect of the NLRP3 inflammasome on the hippocampus pathology after behavioral experiments (day 46). As shown in Fig. 3a, EAE mice, compared with control mice, exhibited irregular distribution of hippocampal neurons, cytoplasmic shrinkage, and triangulated pyknotic nuclei in DG regions. However, MCC950-treated mice showed a reduction in neuronal loss (Fig. 3b).

To verify the structural plasticity, we measured the alterations in dendrite complexity and spine density in the hippocampal DG region using Golgi staining. Neurons in the hippocampal region were sparse and disorderly in the EAE mice, while MCC950 pretreatment ameliorated the hippocampal pathology (Fig. 3c, d). By concentric circle (Sholl) analysis, we found that the neurite arborization (Fig. 3e) and the spine density (Fig. 3f) were significantly reduced in the EAE mice compared with the control mice, whereas pretreatment with MCC950 alleviated this decrease (Fig. 3e, f).

At the molecular level, we detected the levels of the postsynaptic marker PSD95 and the presynaptic marker synapsin І in hippocampal tissue by western blotting and immunohistochemistry. The expression of PSD95 and synapsin І were remarkably reduced in EAE mice, while pretreatment with MCC950 restored the levels of these protein (Figs. 3g and S6). The data above indicate that MCC950 treatment can ameliorate hippocampal pathology.

### Activation of NLRP3 inflammasome induces astrocyte conversion to A1 phenotype

**Fig. 4:**
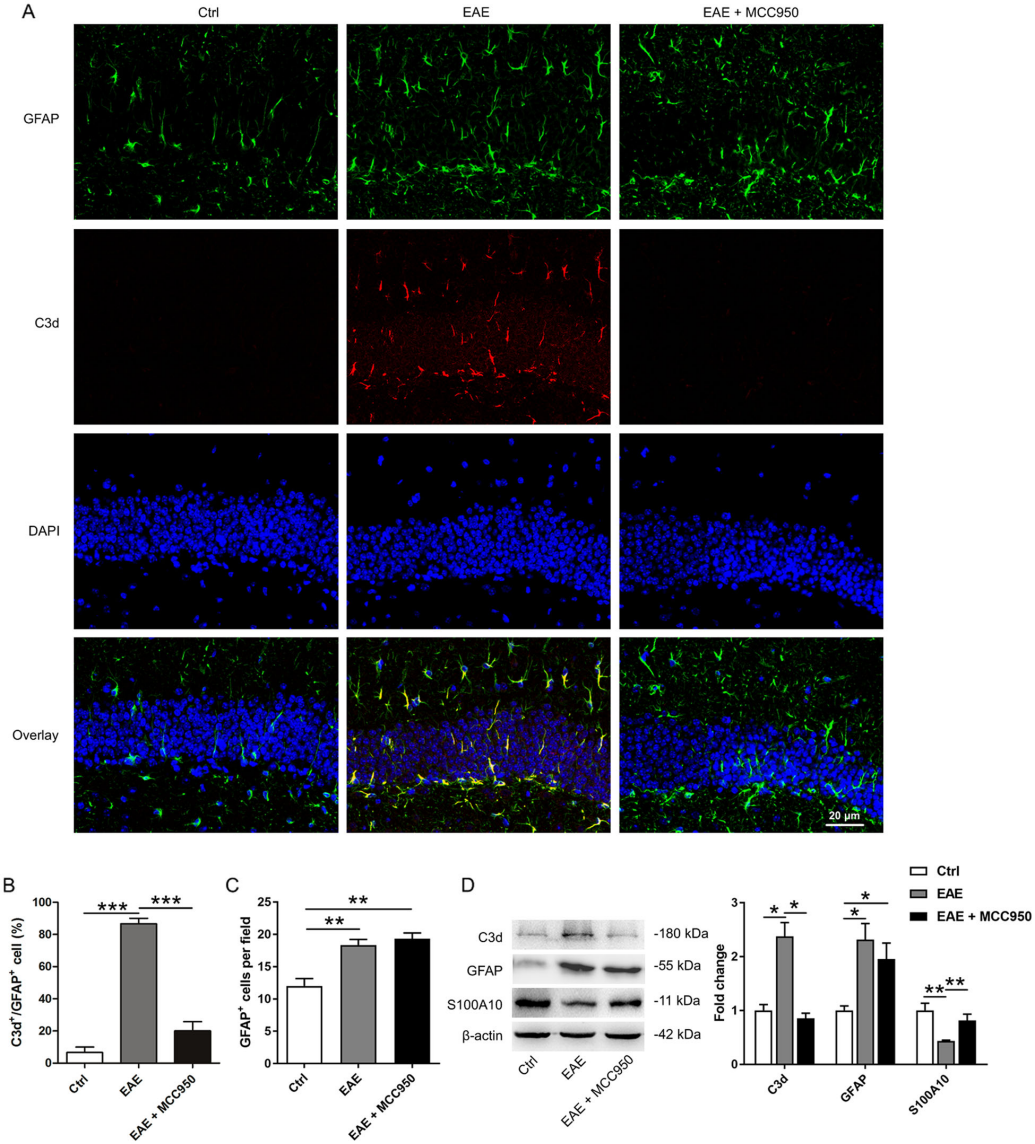
Activation of the NLRP3 inflammasome induces astrocyte conversion to A1 phenotype. **a** The co-localization of C3d (red) and GFAP (green) was assessed by confocal microscopy. **b** The percentage of GFAP-positive astrocytes that were C3d-positive in the hippocampus DG region. **c** The quantification of GFAP-positive astrocytes in the hippocampus DG region. Field: 70 × 70 μm. **d** Western blots and densitometric analysis for C3d, GFAP, and S100A10. β-actin was used as an internal control. The data are the mean ± SEM, *n* = 6 biologically independent animals, *P < 0.05, **P < 0.01, ***P < 0.001.

It has been reported that reactive astrocytes in MS patients are complement component 3 (C3)-expressing A1 astrocytes^18^. Therefore, we performed immunohistochemistry (Fig. 4a) and western blotting (Fig. 4d) to assess the change of astrocyte phenotypes in the EAE model. We found that the number of C3d (A1 reactive astrocytes marker) positive astrocytes and the level of C3d were increased in the EAE mice, whereas this increase was blocked by MCC950 (Fig. 4a, b, d). Interestingly, after MCC950 treatment, we found an increased number of GFAP-positive cells compared with that in the control group (Fig. 4a, c). Therefore, we tested the protein level of the A2 reactive astrocytes marker S100A10. We found that the expression of S100A10 was remarkably reduced in the hippocampus of the EAE mice, while pretreatment with MCC950 increased the expression of S100A10 (Fig. 4d). Taken together, these results indicate that the activation of NLRP3 inflammasome in microglia might induce astrocytes conversion to neurotoxic A1 phenotype. NLRP3 inflammasome regulates astrocyte phenotype conversion.

### IL-18 is sufficient to induce astrocyte conversion to the A1 phenotype

**Fig. 5:**
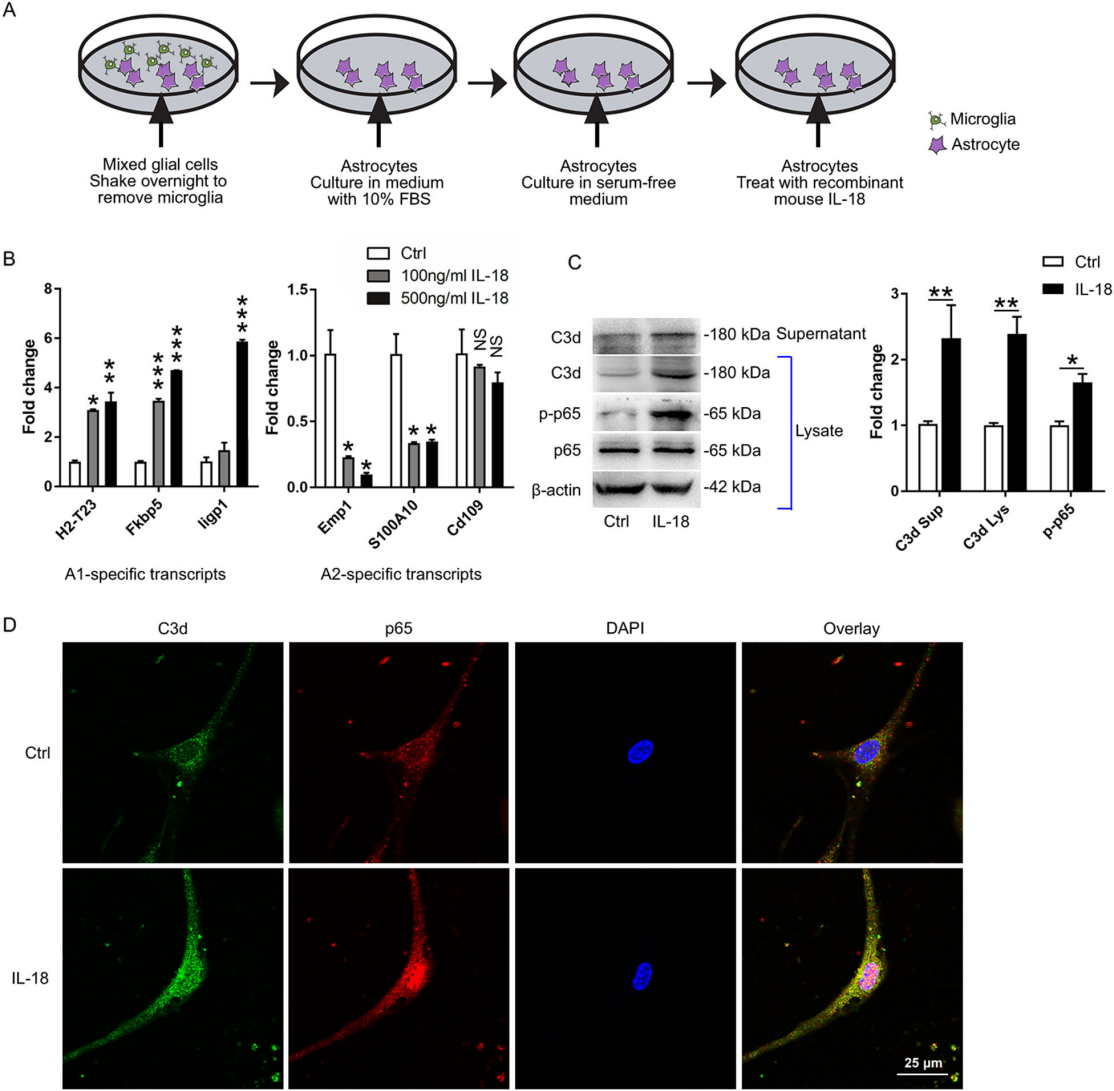
IL-18 is sufficient to induce astrocyte conversion to the A1 phenotype. **a** A schematic diagram of primary astrocytes treatment with IL-18. **b** qPCR analysis of cultured astrocytes treated with IL-18 (A1 marker: *H2-T23*, *Fkbp5 and ligp1*; A2 marker: *Emp1*, *S100A10*, and *Cd109*). **c** Western blots and densitometric analysis for C3d, p-p65, and p65. The relative expression of C3d normalized to β-actin and the relative expression of p-p65 normalized to p65. **d** Immunofluorescence for C3d (green) and p65 (red) in cultured astrocytes. The data are the mean ± SEM, *n* = 6, *P < 0.05, **P < 0.01, ***P < 0.001.

The activated NLRP3 inflammasome processes pro-IL-1β and pro-IL-18 to produce mature IL-1β and IL-18, respectively. IL-1β and IL-18 have similar properties, such as, an inactive precursor, activation by danger associated factors and similar signaling events^33,34^. Elevated levels of IL-18 in the serum and cerebrospinal fluid (CSF) have been reported in MS patients^35,36^. However, there are conflicting reports regarding the levels of IL-Iβ in the CSF of MS patients^37–39^. Moreover, the IL-18 receptor and GFAP are colocalized in astrocytes^11,40,41^. Additionally, we found that the levels of C3d in astrocytes were not increased upon treatment with IL-1β (Fig. S7B), which is consistent with the previous research^18^. Therefore, primary mouse astrocytes were cultured and were treated with 100 ng/ml or 500 ng/ml recombinant mouse IL-18 (Fig. 5a). The qPCR results showed that the levels of the A1-specific gene were increased, and the levels of the A2-specific gene were reduced after 500 ng/ml IL-18 treatment (Fig. 5b). Therefore, the concertation of 500 ng/ml IL-18 was used for subsequent experiments. We found that the levels of C3d in both astrocyte supernatant and astrocyte lysate were increased upon treatment with IL-18 (Fig. 5c). It has been reported that A1 reactive astrocytes are induced by nuclear factor kappa beta (NF-κB) pathway^42^. We found an increased level of p-NF-κB p65 (active form) after IL-18 treatment (Fig. 5c). Consistent with the western blotting data, the immunofluorescence results also revealed that treatment with IL-18 significantly increased the expression of C3d and induced the translocation of NF-κB from cytoplasm to nuclei (Fig. 5d). Taken together, these results indicate that IL-18 induces astrocyte conversion to the A1 phenotype through NF-κB pathway.

As the accumulation of C3 increase the neuronal damage^42^, we determined whether C3d affected with neuron in EAE mice. Increased C3d was found around the MAP2-positive neuron in EAE mice, while pretreatment with MCC950 decreased the accumulation of C3d (Fig. S8). These results indicate the possible functional role for C3d at neuronal damage in EAE mice.

### IL-18-induced A1 reactive astrocytes impair hippocampal neurons through the release of C3

**Fig. 6:**
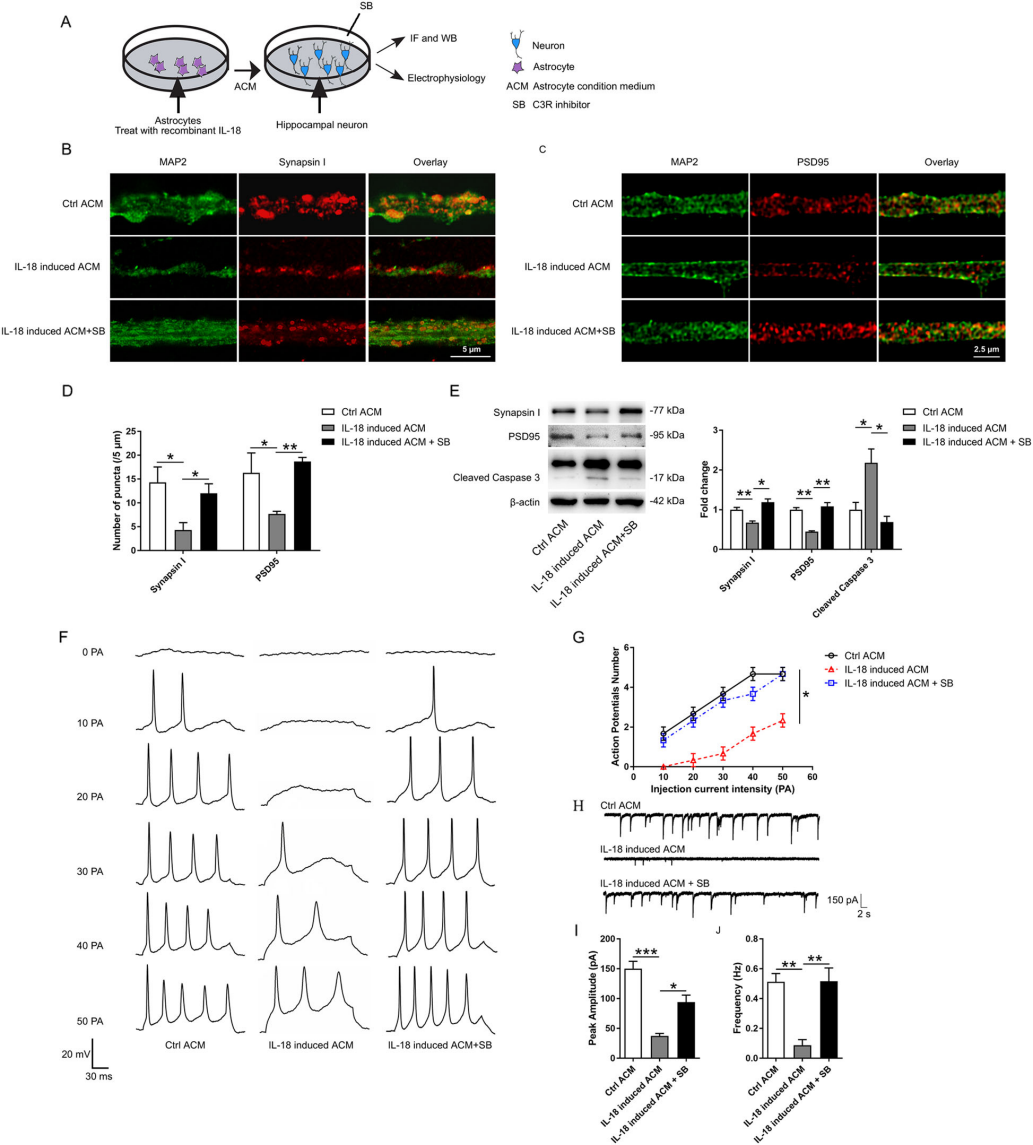
IL-18-induced A1 reactive astrocytes impair hippocampal neurons through the release of C3. **a** A schematic diagram of hippocampal neurons treatment with different astrocyte conditioned medium (ACM). **b**–**d** Representative images and the quantification of immunostaining for synapsin I and PSD95 in cultured neurons treated with control ACM, IL-18 induced ACM or IL-18 induced ACM + SB290157. **e** Western blots and densitometric analysis for synapsin I, PSD95, and cleaved caspase-3 in cultured neurons. β-actin was used as an internal control. **f** Representative traces of whole-cell patch clamp evoked AP recordings from cultured neurons. **g** The quantification of APs evoked with different current injections from 0 to 50 pA with a duration of 200 ms. **h** Representative traces of whole-cell patch clamp sEPSC recordings from cultured neurons. **i** Quantification of sEPSC amplitude. **j** Quantification of sEPSC frequency. The data are the mean ± SEM, *n* = 6, *P < 0.05, **P < 0.01, ***P < 0.001.

Normally astrocytes promote CNS neuronal survival, whereas A1 reactive astrocytes decrease the synaptic density and impair synaptic function through the release of C3^18,42^. To test whether IL-18 induced A1 reactive astrocytes can impair the synaptic density through C3, primary hippocampal neurons were cultured in different ACM, and the density of the synapses was quantified by immunostaining and western blotting with pre- and postsynaptic markers (Fig. 6a). Hippocampal neurons cultured with IL-18 induced ACM showed a significantly decreased synaptic density compared to that of hippocampal neurons cultured with control ACM, whereas pretreatment with C3R inhibitor (SB290157) alleviated this decrease (Fig. 6b–e). We also investigated the effect of IL-18 induced A1 reactive astrocytes on neuronal apoptosis using caspase-3 antibody. The western blotting data showed that, compared with control ACM, IL-18 induced ACM promoted neuronal death, and SB290157 treatment significantly reduced the neuronal death (Fig. 6e). To further determine the effects of IL-18 induced A1 reactive astrocytes on synaptic function, we performed whole-cell patch clamp recording on hippocampal neurons cultured in different ACM. Compared with hippocampal neurons cultured with control ACM, those neurons cultured with IL-18 induced ACM exhibited a significantly decreased frequency of evoked APs (Fig. 6f, g). Moreover, hippocampal neurons cultured with IL-18 induced ACM also exhibited a significantly decreased frequency and amplitude of sEPSCs compared to those of neurons cultured with control ACM (Fig. 6h–j). Additionally, pretreatment with SB290157 decreased the evoked APs and sEPSCs.

In order to eliminate the possibility that the residual IL-18 in ACM had some effect on neurons. After treating astrocytes with IL-18 for 24 h, we investigated the level of remained IL-18 in ACM by ELISA. We found that the level of IL-18 was too low to be measured by ELISA (Fig. S9A). Primary hippocampal neurons were treated with PBS or recombinant mouse IL-18 (100 ng/ml and 500 ng/ml, R&D Systems) for 24 h, and the levels of synapsin I and PSD95 were measured by western blotting. No significant difference was found in these groups (Fig. S9B).

Taken together, these results show that IL-18 induced A1 reactive astrocytes impair hippocampal neurons through the release of C3.

## Discussion

MS patients are often affected with cognitive deficits, and cognitive deficits occur from clinically isolated syndrome (CIS) to relapsing MS, or even progressive MS^4,43–47^. The pattern of cognitive deficits in MS is characterized by impairment in sustained attention, verbal and nonverbal memory, conceptual reasoning, and information processing speed, while language and intellectual functions are preserved^43,48^. Currently, there is no proven effective treatment for MS-related cognitive deficits. EAE mice are used to model the disease progression of MS and mirror MS-like pathology. It has been reported that the EAE mice show memory impairments, as tested by fear conditioning test, before they exhibit motor disabilities^8^. Moreover, glial cells are necessary for hippocampal synaptic alterations and contextual learning-memory impairment in EAE^6,8^, which may induce EAE-related cognitive deficits.

The NLRP3 inflammasome are implicated in several inflammatory diseases and plays a key role in gliosis^49,50^. Some studies have indicated that NLRP3 inflammasome activation is involved in mediating synaptic dysfunction, cognitive impairment, and microglial dysfunction in AD models, and that the inhibition of the NLRP3 inflammasome attenuates spatial memory impairment and enhances Aβ clearance in AD models^29,51,52^. However, there is no research on NLRP3 inflammasome in MS-related cognitive deficits. In our study, we found that microglia and NLRP3 inflammasome were activated in the hippocampus of EAE mice, while pretreatment with MCC950 inhibited the activation of microglia and NLRP3 inflammasome (Fig. 1). Key factors in the inflammasome pathway were also assessed. The levels of pro-IL-1β, IL-18, ASC, and cleaved caspase-1 p10 were increased, and pretreatment with MCC950 decreased the expression of these proteins (Figs. 1c and S1C). In our experiment, we have found the difference in pro-IL-1β between different groups. However, we could hardly measure the cleaved form of IL-1β using many different brands of IL-1β antibodies (Fig. S1C). We thought this might because the level of mature IL-1β is very low in the brain^39^. Moreover, we assessed the effect of NLRP3 inflammasome on EAE-related cognitive deficits. Previous studies have reported that EAE mice show learning-memory impairment in the late phase by long-term potentiation (LTP)^32^, which is consistent with our behavioral results. Furthermore, our behavioral data showed that EAE-induced cognitive deficits could be ameliorated by MCC950 treatment (Fig. 2). It has been reported that neuronal apoptosis and synapse loss are found in the hippocampus of EAE mice^5–7^, which is consistent with our morphological results. Furthermore, we found that inflammasome inhibition improved hippocampal pathology in EAE mice (Fig. 3). Taken together, our results demonstrate that the NLRP3 inflammasome participates in the pathogenesis of cognitive deficits in EAE animals, and that pretreatment with an NLRP3 inhibitor could significantly ameliorate EAE-related cognitive deficits and reduce the hippocampal pathology.

IL-1β and IL-18 are inflammatory effector cytokine processed by NLRP3 inflammasomes. It has been reported that IL-1β could stimulate some cells to release cytokines and produce free radical NO, leading to neurotoxicity^53^. IL-18 could also modulate neuronal excitability, and inhibit long-term-potentiation, a form of a neuronal plasticity considered to underlie learning and memory^54^. However, few studies have investigated the effects of IL-1β and IL-18 on astrocytes.

In many brain disorders, microglia activation occurs before astrogliosis. The inhibition of NLRP3 inflammasome significantly reduces the activation of microglia and alters the activation of astrocytes^55–61^. It is now clear that the reactive astrocytes exist in at least two different states, the A1 (neurotoxic) state and the A2 (neuroprotective) state^18^. In fact, A1 reactive astrocytes are present in the brains of some neurodegenerative diseases (such as PD, HD, AD, ALS, and MS), and C3 is one of the most characteristic and highly upregulated markers of A1 reactive astrocytes^18^. In our study, C3d was highly expressed in the hippocampal astrocytes of EAE mice, which is consistent with the results of previous study on A1 reactive astrocytes in MS patients^18^. We also found that pretreatment with MCC950 reduced the expression of C3d and upregulated the expression of S100A10 (an A2 astrocyte marker) (Fig. 4). These results indicate that astrocytes are converted to neurotoxic A1 reactive astrocytes in EAE mice and this process is regulated by the NLRP3 inflammasome.

Microglia-derived inflammatory factors has important effects on A1 astrocyte reactivity^18,61^. Liddelow found that IL-1α, TNFα, and C1q, which are secreted by LPS-activated microglia, can induce A1 reactive astrocytes^18,62^. Regarding the NLRP3 inflammasome, MCC950 only inhibits the secretion of IL-1β and IL-18 from macrophages or microglia, but does not block TNFα and IL-1α^24^. Therefore, it was interesting to identify the factor that induces A1 reactive astrocytes in our experiment. IL-18, a member of the IL-1 pro-inflammatory cytokines family, diversely regulates immunity and inflammatory responses in inflammatory-associated disorders. The canonical action of IL-18 occurs via the recruitment of the adapter myeloid differentiation factor (MyD88). This event leads to the nuclear translocation of NF-κB and the subsequent modulation of transcription^63^. NF-κB-activated astrocytes are associated with neuroinflammation and may represent harmful astrocytes that promote neurodegeneration^42,64–66^. Regarding IL-1β, it has been reported that IL-1β is unable to induce the expression of A1 transcripts^18^. Therefore, in our experiment, primary mouse astrocytes were cultured and treated with recombinant mouse IL-18. The results showed that IL-18 is sufficient to induce astrocyte conversion to the A1 phenotype (Fig. 5). We also found that IL-18 induced A1 reactive astrocytes can impair hippocampal neurons by secreting C3 (Fig. 6). Our results suggest that IL-18 may act as a new inducible factor for A1 reactive astrocytes.

In summary, our data show that the NLRP3 inflammasome is activated and contributes to cognitive deficits in EAE mice, and it may act via regulating astrocyte phenotype alteration. Our study might reveal the mechanisms of cognitive deficits and provides a novel therapeutic strategy for hippocampal impairment in MS.

## Supplementary information

Figure S1

Figure S2

Figure S3

Figure S4

Figure S5

Figure S6

Figure S7

Figure S8

Figure S9

Legends of Supplemental figures

## Data Availability

The data that support the finding of this study are available upon request from the corresponding author.
