# Supplementary material for: Inhibition of the NLRP3-inflammasome prevents cognitive deficits in experimental autoimmune encephalomyelitis mice via the alteration of astrocyte phenotype
Source: Cell Death Dis. 2020 May 15;11(5):377. doi: 10.1038/s41419-020-2565-2 (PMC7229224; doi:10.1038/s41419-020-2565-2)
Supplement: Supplementary file 10 — Legends of Supplemental figures [file 41419_2020_2565_MOESM10_ESM.docx]

**Legends of Supplement Figures**

Fig. S1 (A) Immunofluorescence for ASC (green) and DAPI (blue) in the hippocampus of each group. (B) The number of ASC specks in the hippocampus of each group. Field: 200 × 200 μm. (C) Western blot and densitometric analysis for pro-IL-1β and CD68. β-actin was used as an internal control. The data are mean ± SEM, n = 6, **P* < 0.05, ** *P* < 0.01, *** *P* < 0.001.

Fig. S2 Immunohistochemistry for F4-80 positive cells in the hippocampus and spinal cord of EAE mice.

Fig. S3 (A) MCC950 significantly reduced the EAE clinical scores. (B) The body weight had no significant difference between each group. The data are the mean ± SEM, n = 15.

Fig. S4 MCC950 prevented demyelination in the EAE mice. Luxol fast blue (A) and mature oligodendrocyte marker (MBP) (B) staining of the lumbar spinal cord in each group on day 19 after EAE induction. (C) Representative TEM images myelin in the lumbar spinal cord of each group on day 19 after EAE induction. The data are the mean ± SEM, n = 6, **P* < 0.05, ** *P* < 0.01, *** *P* < 0.001.

Fig. S5 Representative TEM images myelin in the lumbar spinal cord of each group on day 40 after EAE induction. The data are the mean ± SEM, n = 6.

Fig. S6 Immunohistochemistry for PSD95 and Synapsin I in the hippocampus of each group on day 40 after EAE induction. The data are the mean ± SEM, n = 6, ** *P* < 0.01, ****P* < 0.001.

Fig. S7 (A) qPCR analysis of cultured astrocytes treated with IL-1β (A1 marker: *H2-T23*, *Fkbp5* and *ligp1*; A2 marker: *Emp1*, *S100A10* and *Cd109*). (B) Western blots and densitometric analysis for C3d and S100A10. β-actin was used as an internal control. The data are mean ± SEM, n = 6, **P* < 0.05, ** *P* < 0.01.

Fig. S8 Representative images of immunostaining for MAP2 (red) and C3d (green) in the neurons of each group on day 40 after EAE induction.

Fig. S9 (A) IL-18 could not be detected in IL-18 induced ACM by ELISA. (B) Western blot and densitometric analysis for Synapsin [I](https://www.ncbi.nlm.nih.gov/gene/6853) and PSD95. β-actin was used as an internal control. The data are mean ± SEM, n = 6.
